# Impact of Social Support on Cardiovascular Risk Prediction Models: A Systematic Review

**DOI:** 10.7759/cureus.45836

**Published:** 2023-09-24

**Authors:** Mansi Singh, Aiswarya Nag, Lovish Gupta, Jingle Thomas, Rakshana Ravichandran, Binay K Panjiyar

**Affiliations:** 1 Medicine, O.O. Bogomolets National Medical University, Kyiv, UKR; 2 Internal Medicine, Sri Ramachandra Institute of Higher Education and Research, Chennai, IND; 3 Internal Medicine, Maulana Azad Medical College, New Delhi, IND; 4 Internal Medicine, Al-Ameen Medical College, Vijayapura, IND; 5 Internal Medicine, Rajarajeswari Medical College and Hospital, Bangalore, IND; 6 Department of Internal Medicine, Harvard Medical School, Boston, USA; 7 Internal Medicine, California Institute of Behavioral Neurosciences & Psychology, Fairfield, USA

**Keywords:** social determinants of health, sdoh, social isolation, loneliness, social life, cardiovascular risk prediction models, risk prediction model, cardiovascular risk factors, cardiovascular diseases, social support

## Abstract

Cardiovascular diseases (CVD) stand as the primary causes of both mortality and morbidity on a global scale. Social factors such as low social support can increase the risk of developing heart diseases and have shown poor prognosis in cardiac patients. Resources such as PubMed and Google Scholar were searched using a boolean algorithm for articles published between 2003 and 2023. Eligible articles showed an association between social support and cardiovascular risks. A systematic review was conducted using the guidance published in the Cochrane Prognosis Method Group and the PRISMA checklist, for reviews of selected articles. A total of five studies were included in our final analysis. Overall, we found that participants with low social support developed cardiovascular events, and providing a good support system can decrease the risk of readmission in patients with a history of CVD. We also found that integrating social determinants in the cardiovascular risk prediction model showed improvement in accessing the risk. Population with good social support showed low mortality and decreased rate of readmission. There are various prediction models, but the social determinants are not primarily included while calculating the algorithms. Although it has been proven in multiple studies that including the social determinants of health (SDOH) improves the accuracy of cardiovascular risk prediction models. Hence, the inclusion of SDOH should be highly encouraged.

## Introduction and background

Among the world's top causes of death and many significant medical disorders are cardiovascular diseases (CVD) [1]. Risk factors causing CVD include age, gender, obesity, genetics, smoking history, hypertension, diabetes, socio-economic status, lifestyle, etc [2]. Timely identification of risks in individuals is necessary as CVD advances over time, and waiting until the symptoms occur increases morbidity and mortality [3].

People with poor social health were 30% more likely to develop coronary heart disease (CHD) and stroke episodes, according to a systematic review published in 2016 constituting 23 studies [4]. The notion of "social health" refers to an individual's potential for fulfilling and significant acquaintances, their ability to adapt to social adjustment, and their interactions with and perception of support from other people, organizations, and services.

**Figure 1:**
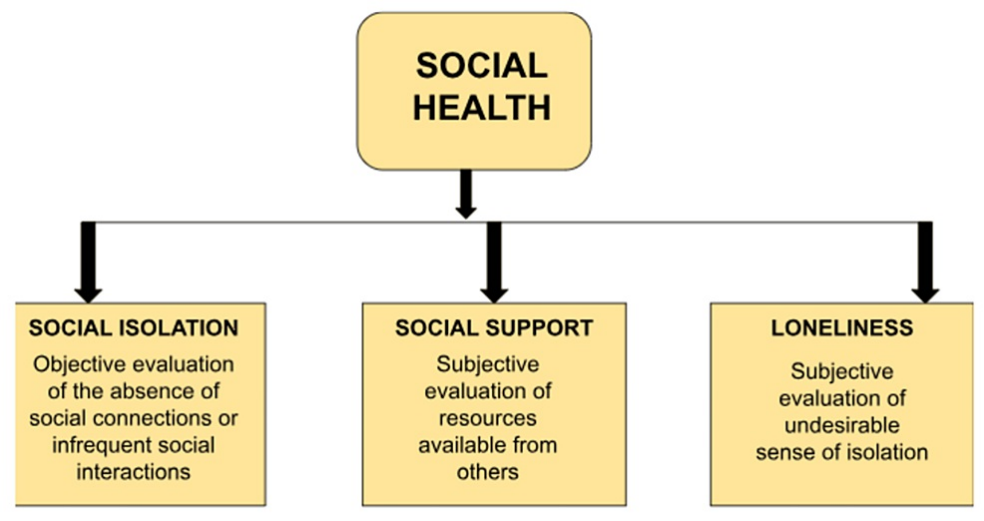
Components of social health

The terms social isolation, social support, and loneliness are frequently used to describe social health, more briefly described in Figure 1. Social support is a subjective indicator of an individual’s perception of how much they receive from others [4]. Several studies have shown a relation between social support and change in lifestyle, which can decrease the risk of CVD. While patients with low social support tend to show poor prognosis in cardiac diseases [5]. Some studies also showed the risk of mortality and readmission in patients who are already discharged after hospitalization for heart failure [6,7].

Many risk prediction models and algorithms are being developed to assess the risks of developing CVD, such as systematic coronary risk evaluation (SCORE) [8], cardiovascular risk score (QRISK2) [9-11], Framingham [12], and Reynolds risk score [13]. However, none of them focuses solely on the social determinants. We have several data and literature that show us the association between CVD and other risk factors, such as diabetes, hypertension, age, family history, depression, etc. However, at present, we have very limited data and literature that focus on social factors. As we age, our social health becomes poor, which is influenced by several factors. As we get older, we tend to have less social interaction, network, and activities. We rely more on friends and families for emotional support. The loss of a loved one can lead to stress, isolation, and loneliness. By considering these determinants in conjunction with clinical data, healthcare professionals can have a more holistic approach to determining the risks.

Understanding the impact of social support on cardiovascular disease risk prediction models is essential in order to develop more comprehensive and effective strategies for reducing inequalities in cardiovascular health outcomes.

## Review

Methods

We followed the recently published guide from the Cochrane Prognosis Method and used the PRISMA [14] checklist for the study of prediction models.

Search Strategy 

A systematic review of articles was conducted using the reports of the association between social support and cardiovascular risks from 2003 to 2023. Search engines such as PubMed, Google Scholar, and Medline were used. We included the following terms in our searches on August 01, 2023, using the boolean algorithm - (social impact) OR (social support) AND (cardiovascular risk prediction model) OR (heart disease risk prediction model) OR (risk prediction model) OR (cardiovascular abnormalities) OR (risk prediction model), as described in Table 1.

**Table 1:** Search strategy, search engines used, and number of results displayed

Database	Search Strategy	Search Results
1. PubMed	((((((social impact[Title/Abstract]) OR (social support[Title/Abstract])) AND (cardiovascular risk prediction model[Title/Abstract])) OR (heart disease risk prediction model[Title/Abstract])) OR (risk prediction model[Title/Abstract])) OR (cardiovascular abnormalities[MeSH Terms])) OR (risk prediction model[MeSH Terms])	389
2. Google Scholar	(social impact) OR (social support)) AND (cardiovascular risk prediction model)) OR (heart disease risk prediction model)) OR (risk prediction model)	18,400

*Eligibility Criteria* 

We included the articles published in English between 2003 and 2023 with only free full text and studies, including clinical trials, randomized controlled trials, and systematic reviews. The studies consisting of non-randomized controlled trials, books, and documents with a population of age less than 19 years old were excluded, as described in Table 2.

**Table 2:** Inclusion and exclusion criteria adopted during the process of literature search

	Inclusion	Exclusion
Species	Human	Animals
Gender	All	-
Age	Adult: 19+ years	< 19 years old
Types of studies	Clinical trials, randomized controlled trials, systematic reviews	Non-randomized controlled trials, books, and documents
Text availability	Free full-text	Abstract, Free Text
Publication year	2003-2023	Before 2003
Language	English	Non-English

*Screening and Quality Appraisal* 

The articles were reviewed for duplicates using Endnote [15] and thoroughly screened by two authors AN and LG to be included in the final analysis. We employed a range of quality assessment instruments to guarantee the reliability of the five chosen papers. In the case of systematic reviews and meta-analyses, we adhered to the PRISMA checklist. For randomized clinical trials, we utilized the Cochrane bias assessment tool. The quality appraisal of qualitative studies was conducted using critical appraisal skills, as shown in Table 3.

**Table 3:** Quality appraisal tools used PRISMA: Preferred Reporting Items for Systematic Reviews and Meta-Analyses, RCT: Randomized Controlled Trials

Quality Appraisal Tools Used	Type of Studies
PRISMA checklist	Systematic reviews and meta-analysis
Cochrane bias tool assessment	RCT

Results

**Figure 2:**
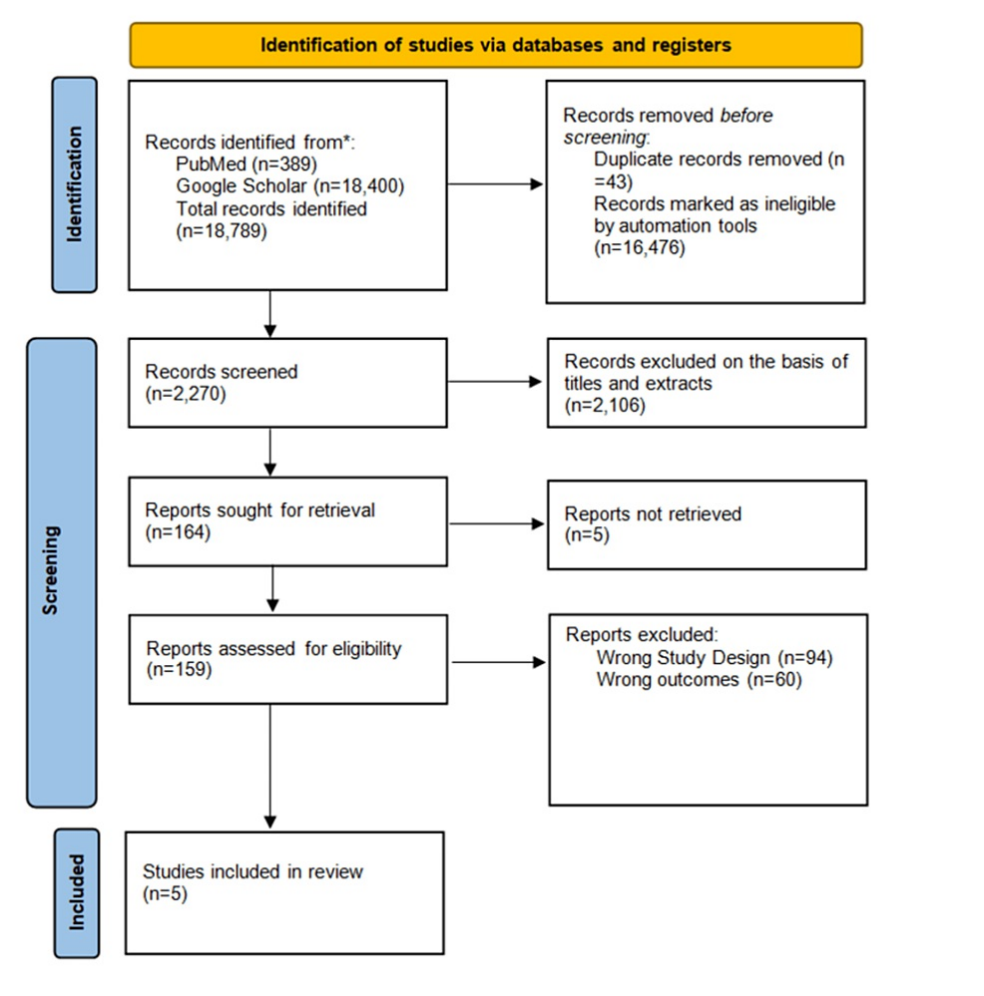
PRISMA 2020 flow diagram for new systematic review showing database searches PRISMA: Preferred Reporting Items for Systematic Reviews and Meta-Analyses

A total of 18,789 articles were identified, including 389 articles from PubMed and 18,400 articles from Google Scholar. However, only five studies met the inclusion criteria, as discussed in Figure 2. Out of these, three studies focused on the impact of support on CVD, and two studies focused on cardiovascular risk prediction models.

In Table 4, we discussed that, in populations with low social support, the risk of developing CVD is high.

**Table 4:** Studies showing the development of cardiovascular events in a low social support population

Study	Participants	Social Support Status	Key Findings
Freak-Poli et al. (2021) [16]	11,486	People with high social support = 11,258, low social support = 228	Cardiovascular disease events in high social support = 1,388 (12.32%), low social support =56 (24.56%)
Shen et al. (2004) [17]	142	Social support provided post-myocardial infarction	Social support impacts directly and indirectly on cardiac health by decreasing depressive symptoms among patients post-infarction
Thurston et al. (2009) [18]	3,003	The incidence of cardiovascular diseases was assessed in a cohort of a population with low social support and loneliness	237 non-fatal vs. 120 fatal cases of cardiovascular diseases observed

Freak-Poli et al. [16] showed that, in a cohort of 11,486 participants (11,258 with high social support and 228 with low social support), 12.32% with high social support and 24.56% with low social support developed the events of CVD. Shen et al. [17] suggested that providing a better social support system decreased depression in participants, which in turn decreased the risk of readmission. Thurston et al. [18] also showed that, in a cohort of 3,003 participants with low social support, 237 participants developed non-fatal vs. 120 participants who developed fatal CVD in the future.

All three studies showed that, in a population with low social support, the chances of developing CVD are higher through multiple pathways, including its impact on stress, behaviors, medication adherence, and mental health.

Table 5 shows the results of integrating social determinants of health (SDOH) with cardiovascular risk prediction models. Both studies showed improvement in the accuracy of the cardiovascular risk prediction model with the integration of SDOH.

**Table 5:** Studies showing the integration of social determinants of health with cardiovascular risk prediction models

Study	Study Design	Participants	Social Determinants Included	Key Findings	Conclusion
Hammond et al. (2020) [19]	Retrospective observational study	4,703	Socio-economic status, access to care, neighborhood environment, habits and behavior (Alcohol usage), social support, education level	Population with low social support(unmarried) are more likely to develop the risk of cardiovascular diseases	Including the social determinants of health (SDOH) with the cardiovascular risk prediction model improved the accuracy
Hammond et al. (2019) [20]	Cohort study	155,013	Socio-economic status, ethnicity	SCORE2 prediction model underpredicted the risk in low socioeconomic subgroups and populations with Surinamese ethnicity	It is necessary to implement social determinants in the cardiovascular risk prediction model

Discussion

This systematic review sheds light on the impact of social support on cardiovascular risk prediction models. At first, we compared different risk prediction models and their components in Table 6 and found out that the social determinants need to be integrated while using these risk prediction models.

**Table 6:** Cardiovascular risk prediction models and their components AF: Atrial Fibrillation, BMI: Basic Metabolic Index, BP: Blood Pressure, CAD: Coronary Artery Diseases, CKD: Chronic Kidney Diseases, CVD: Cardiovascular Diseases, DM: Diabetes Mellitus, HDL: High-Density Lipid, HTN: Hypertension, MI: Myocardial Infarction, RA: Rheumatoid Arthritis

Risk Prediction Models	Components	Features
European systemic coronary risk evaluation algorithm (SCORE)	Age, gender, total cholesterol, systolic BP, smoking status	Estimates the risk of fatal CVD over 10 years
QRISK2	Age, gender, systolic BP, ethnicity, smoking status, ratio of total cholesterol: HDL cholesterol, BMI, history of CAD, treated HTN, RA, CKD, type II DM, AF	Estimates lifetime risk of adverse events of CVD
Framingham	Age, gender, systolic BP, smoking status, total cholesterol	Estimates the risk of CAD over 10 years
Reynolds risk score (RSS)	Age, gender, systolic BP, Total cholesterol, HDL cholesterol, HS-C reactive protein, history of MI	Estimates risks of having MI, stroke, and major CVD in the next 10 years

Our first finding was to establish a link between social support and CVD. In one of the studies, it was noted that 12.32% of the population with high social support and 24.56% of the population with low social support developed the events of CVD in their lifetime. Meanwhile, the second study showed that, in a cohort of 3,003 participants with low social support, 357 participants developed CVD.

Our second and most significant finding was that people with low social support and loneliness are more prone to develop depression, and depression can act as an indirect cause of CVD. We can decrease the incidence of re-hospitalization in patients with post-infarction by providing them with more support. Social support systems can contribute to reducing stress in the population. Stress can directly lead to hypertension. Social support can also influence lifestyle, such as smoking, diet, etc. Hence, positive social support has a positive effect on risk management and directly impacts the development of CVD.

The impact of social support on cardiovascular risk prediction models stands as a topic of paramount significance. Extensive research underscores the substantial influence of social support on cardiovascular health and risk factors. Numerous studies have ascertained that individuals possessing robust social support networks tend to experience more favorable cardiovascular outcomes and exhibit a reduced likelihood of developing CVD. To elaborate, social support has been associated with shortened hospital stays, enhanced treatment adaptation, and a diminished risk of mortality among patients dealing with chronic conditions [6,7].

Moreover, it is worth noting that inadequate social and economic resources, insufficient social support, and social isolation have all emerged as recognized risk factors contributing to the development of coronary heart disease. Social support manifests in various forms, encompassing instrumental, emotional, and informational support. Research findings consistently underscore the pivotal role that social support plays in mitigating the risk of CVD and enhancing overall cardiovascular well-being.

Our third finding focused on integrating SDOH with cardiovascular risk prediction models, and we found out that incorporating SDOH with these risk prediction models has increased their accuracy. SDOH are the conditions and components in the settings where individuals are born, live, learn, work, play, and age that have an impact on a variety of hazards and repercussions for health, functioning, and quality of life. To put it differently, social and economic issues have an effect on people's health and well-being both individually and collectively.

SDOH includes various elements such as economic stability, social and economic factors, education, surroundings, and access to healthcare.

Economic stability: This entails aspects such as earnings, employment, and access to necessities like food and housing. Health disparities are more likely to affect people with lower incomes or undetermined employment.

Social and economic factors: Income inequality, social cohesion, and discrimination are a few examples of social and economic issues that might have an impact on health.

Education: The availability of and achievement in schooling can influence health outcomes. Better health and well-being are frequently associated with higher levels of education.

Surrounding: Health can be impacted by the physical surroundings in which individuals reside, including elements such as accessibility to green areas, walkability, and exposure to environmental contaminants.

Access to healthcare: While healthcare itself is an important factor, access to healthcare services, the quality of care received, and health insurance coverage all play a role in determining health outcomes.

Therefore, healthcare professionals should consider integrating measures of social support into cardiovascular risk assessment tools to better understand and predict an individual's risk of developing CVD. However, the question arises of how can we move towards this holistic approach of integrating SDOH with cardiovascular risk prediction models. Here are some strategies in Table 7.

**Table 7:** Strategies to integrate SDOH with cardiovascular risk prediction models SDOH: Social Determinants of Health

SDOH Incorporation Strategies	Components
SDOH data	Collect SDOH data of patients either by survey or pre-existing data. These data should include information on income, education, employment status, neighborhood characteristics, access to healthcare, social support networks, and more.
Incorporate variables	Include socioeconomic status (income, education) as covariates in the model. Use neighborhood-level variables like crime rates, access to healthy food, and proximity to parks as proxies for environmental factors. Include measures of social isolation.
SDOH score	Create a composite SDOH score that combines various SDOH variables to represent an individual's overall social and economic context. This score can be integrated into the risk model as a single variable.
Validation	Ensure that the SDOH-integrated risk model is validated and calibrated to accurately predict cardiovascular risk in diverse populations. This may require access to large datasets that include both clinical and SDOH data.
Implementation	Once developed and validated, integrate the SDOH-enhanced risk prediction model into clinical practice.

Integrating SDOH into cardiovascular risk prediction models acknowledges that health outcomes are influenced by a complex interplay of factors beyond traditional clinical risk factors. This approach can help identify individuals at higher risk and guide interventions that address both clinical and social determinants to improve cardiovascular health and reduce health disparities.

Limitations

Our systematic review has certain constraints. We exclusively considered articles published in the English language within the two past decades. Our selection was limited to papers accessible for free on PubMed and Google Scholar. Additionally, we focused solely on studies involving individuals aged 19 years and older, and our search was restricted to papers addressing cardiovascular risk prediction models and social health. Due to less research in this particular area, there were limitations in articles retrieved for final analysis. To obtain more precise findings, further research is required.

## Conclusions

The implications of increased social support are profound. Increased social support has been linked to lower levels of loneliness, perceived stress, and anxiety, as well as better mental and physical health. Additionally, increased social support has been found to reduce the odds of depressive and psychotic-like symptoms, which is especially important for individuals with severe and persistent mental illness who may be at an increased risk of isolation. Overall, evidence suggests that social support can have a protective effect on cardiovascular health and risk, as well as on overall mental and physical well-being. In light of this research and the evidence presented, it is clear that social support plays a significant role in cardiovascular risk prediction models.
